# Cyclophilin D participates in the inhibitory effect of high‐fat diet on the expression of steroidogenic acute regulatory protein

**DOI:** 10.1111/jcmm.14569

**Published:** 2019-08-01

**Authors:** Xiaohui Su, Dong Lin, Dandan Luo, Mingqi Sun, Xiaolei Wang, Jifeng Ye, Meijie Zhang, Yikun Zhang, Xiaolin Xu, Chunxiao Yu, Qingbo Guan

**Affiliations:** ^1^ Department of Endocrinology, Shandong Provincial Key Laboratory of Endocrinology and Lipid Metabolism Shandong Provincial Hospital affiliated to Shandong University Jinan China; ^2^ General Practice Jinan City People's Hospital Jinan China; ^3^ Peking Union Medical College, Chinese Academy of Medical Sciences Graduate School of Peking Union Medical College Beijing China; ^4^ Shandong Institute of Endocrine & Metabolic Diseases Shandong First Medical University & Shandong Academy of Medical Sciences Jinan China; ^5^ Department of Endocrinology and Metabolism The Second People's Hospital of Liaocheng Liaocheng China; ^6^ Department of Rheumatology and Immunology Dongying People's Hospital Dongying China

**Keywords:** cyclophilin D, high‐fat diet, mitochondrial dysfunctions, steroidogenic acute regulatory protein, testosterone deficiency

## Abstract

**Objective:**

The high‐fat diet (HFD)–induced obesity is responsible for the testosterone deficiency (TD). However, the mechanism remains unknown. Mitochondrial homeostasis is proved to be important for maintaining the function of steroidogenic acute regulatory protein (StAR), the first rate‐limiting enzyme in testosterone synthesis. As the key regulator of mitochondrial membrane permeability, cyclophilin D (CypD) plays a crucial role in maintaining mitochondrial function. In this study, we sought to elucidate the role of CypD in the expression of StAR affected by HFD.

**Methods:**

To analyse the influence of CypD on StAR in vivo and in vitro, mouse models of HFD, CypD overexpression and CypD knockout (*Ppif* ^−/−^) as well as Leydig cells treated with palmitic acid (PA) and CypD overexpression plasmids were examined with an array of metabolic, mitochondrial function and molecular assays.

**Results:**

Compared with the normal diet mice, consistent with reduced testosterone in testes, the expressions of StAR in both mRNA and protein levels in HFD mice were down‐regulated, while expressions of CypD were up‐regulated. High‐fat intake impaired mitochondrial function with the decrease in StAR in Leydig cells. Overexpression of CypD inhibited StAR expressions in vivo and in vitro. Compared with C57BL/6 mice with HFD, expressions of StAR were improved in *Ppif* ^−/−^ mice with HFD.

**Conclusions:**

Mitochondrial CypD involved in the inhibitory effect of HFD on StAR expression in testes.

## 1 INTRODUCTION

Testosterone is the most important androgen for male, exerting important physiological effects.^1^ In adult men, testosterone deficiency (TD, total testosterone <300 ng/dL is most commonly used as the cut‐off values for low testosterone)^2^ not only has adverse effects on sexual and reproductive function^3^ but also negatively affects glucose and lipid metabolism, cardiometabolic functions, body composition, bone mineral density and quality of life.^4^, ^5^, ^6^ The global prevalence of TD in adult men ranges from 10% to 40%; among these, the incidence in the United States varies from 24% to 39%, and it ranges from 17% to 33% in China.^2^ With the economic development and life improvement, people are more and more exposed to long‐time and excessive high‐fat diet (HFD) and sedentary lifestyle, which could increase bodyweight, induce glucose intolerance and insulin resistance, and cause dyslipidaemia.^7^, ^8^ In addition, long‐time HFD has been reported to decrease testosterone levels^9^, ^10^; however, the mechanisms underlying have not yet been fully elucidated.

Testosterone, a steroid hormone, is mainly (95%) synthesized and secreted by the testicular Leydig cells.^11^ In response to luteinizing hormone (LH), testicular testosterone biosynthesis is a multi‐step process in which there are a series of steroidogenic proteins and enzymes, such as the steroidogenic acute regulatory protein (StAR), a cholesterol side‐chain cleavage enzyme (P450scc or CYP11A) and 3β‐hydroxysteroid dehydrogenase (3β‐HSD).^12^, ^13^ StAR has been shown to accelerate the transport of cholesterol, raw materials for testosterone synthesis, from the outer mitochondrial membrane (OMM) to the inner mitochondrial membrane (IMM), which is the first and rate‐limiting step of the testosterone biosynthesis.^14^, ^15^ Both the people suffer from congenital lipoid adrenal hyperplasia (lipoid CAH) caused by mutations in the StAR gene and StAR null mice almost completely fail to synthesize steroids, which indicates that the StAR protein is an indispensable ingredient in the process of steroid synthesis.^16^ A previous study reported that both pharmacological inhibition of ATP synthesis and dissipation of mitochondrial membrane potential (Δψm) could inhibit steroidogenesis owing to the reduced expression of StAR,^17^ proving that the StAR activity requires mitochondrial homeostasis. It is reported that HFD could down‐regulate StAR expression in mice.^18^ However, whether mitochondrial dyshomeostasis is involved in HFD‐induced StAR expression down‐regulation is still unclear.

Transient opening of the mitochondrial permeability transition pore (mPTP), a tunnel‐like multiprotein complex spanning across the OMM and IMM,^19^ in response to normal stimuli is important to maintain mitochondrial homeostasis.^20^, ^21^, ^22^ However, under conditions of extreme detrimental stress, irreversible opening of mPTP leads to mitochondrial dysfunction characterized by the dissipation of Δψm, the uncoupling of oxidative phosphorylation followed with the inhibition of ATP generation, increased generation of reactive oxygen species (ROS), mitochondrial swelling, rupture and death.^21^, ^22^, ^23^ Although molecular components of the mPTP have been still vague to date,^22^ cyclophilin D (CypD), a peptidyl prolyl cis‐trans isomerase F (PPIF) in mitochondrial matrix, has been demonstrated from gene deletion studies to be an indispensable regulator for mPTP opening.^24^, ^25^, ^26^ At present, CypD has been shown to translocate from matrix to the IMM and bind to major component candidates of mPTP, including adenine nucleotide translocase (ANT), phosphate carrier (PiC) and oligomycin sensitivity conferring protein of the FoF1 ATP synthase (OSCP)^24^, ^27^, ^28^ across the IMM, which is the foremost step in the process of facilitating PTP opening in response to various stress stimuli.^23^, ^29^ Xiaolei Wang et al have shown that HFD could elevate CypD protein expression levels and could induce the mitochondrial dysfunction in the mouse liver; meanwhile, CypD ablation could ameliorate HFD‐induced hepatic mitochondrial stress, demonstrating that HFD could cause mitochondrial dysfunction by up‐regulating the CypD expression in liver.^30^ Therefore, we hypothesize that CypD‐dependent mitochondrial dysfunction in Leydig cells of testes is involved in HFD‐induced StAR expression deregulation.

Here, to testify and dissect the hypotheses, the expression of CypD and StAR was determined in vivo and in vitro with different administrations including HFD, CypD overexpression and CypD knockout. Meanwhile, through a series of measurements of mitochondrial function, the modulation of HFD on mitochondria is analysed. In the present study, we report for the first time that up‐regulation of CypD expression levels in Leydig cells of testes induced the mitochondrial dysfunction and participates in the HFD‐induced StAR expression deregulation. CypD might be a new therapeutic target in the high‐fat diet–induced TD.

## 2 MATERIALS AND METHODS

### 2.1 Animals and diets

This study was approved by the Animal Ethics Committee of Shandong Provincial Hospital affiliated to Shandong University. Six‐week‐old C57BL/6 male mice were purchased from Beijing Vital River Laboratory Animal Technology Co Ltd. (Beijing, China) and housed in constant temperature‐controlled rooms with a 12‐hour light/dark cycle. After acclimated for two weeks, the mice were, respectively, fed with either normal diet (ND) including 15 kcal% fat (Beijing Keaoxieli Co. Ltd, China) or HFD including 60 kcal% fat (D12492, Research Diets, Inc, USA). All mice were fed for 16 weeks and weighed every two weeks. After 16 weeks, the mice were killed for the subsequent experiments.

*Ppif* ^−/−^ mice (mice null for *Ppif,* the gene encoding CypD) on the same background with C57BL/6 mice were provided by Dr Heng Du. *Ppif* ^−/−^ mice were backcrossed to the C57BL/6 strain for ten generations to obtain stable *ppif* heterozygosis (HE) male and female mice. Then, C57BL/6 mice (*Ppif^+/+^* mice) and *Ppif* ^−^ *^/^* ^−^ mice in each brood were gained through sexual hybridization of stable HE male and female mice. At the 8th week of age, both these two kinds of mice were fed with either normal diet or high‐fat diet, respectively. After 16 weeks, the mice were killed for the subsequent experiments.

In addition, Ad‐PPIF (CypD overexpression adenovirus) and Ad‐EGFP (empty vector adenovirus) were dissolved in sterile PBS and were injected through the caudal vein to the C57BL/6 mice at the eighth week of age. The adenovirus was given three times within a 6‐day interval. After 3 weeks, these mice were killed for the subsequent experiments.

In the appropriate weeks of age mentioned above, the mice were anaesthetized with pentobarbital sodium and the blood samples were collected from eyes. Simultaneously, the testes were isolated and were preserved in liquid nitrogen rapidly or modified Davidson's fluid (mDF, containing 30% of a 37%‐40% solution of formaldehyde, 15% ethanol, 5% glacial acetic acid and 50% distilled H_2_O) for paraffin sections. Some testes were rapidly cut into pieces of 1 mm × 1 mm × 1 mm sections and then were fixed with 2.5% glutaraldehyde in 0.1 M phosphate buffer for following transmission electron microscope analysis. For paraffin sections, testes preserved in mDF were embedded in paraffin and cut to 5‐μm sections for immunofluorescence. For frozen sections, testes were cut at 5 μm thickness with the microtome portion of the cryostat, which were prepared for the subsequent Oil Red staining.

### 2.2 The culture and treatment of mouse TM3 Leydig cells

The mouse TM3 Leydig cell line was derived from normal immature mouse testis and was purchased from Shanghai Institutes for Biological Sciences, Chinese Academy of Sciences. TM3 cells were cultured in the petri dishes with a 1:1 (v:v) mixture of DMEM:F12 media supplemented with 0.5% antibiotics, 2.5 mmol/L l‐glutamine, 0.5 mmol/L sodium pyruvate, 5% horse serum and 2.5% foetal bovine serum. The cells were cultured in a 5% CO_2_ incubator at 37°C. For detection of the effect of lipid on the process of steroidogenesis in vitro, the cells were co‐cultured with different dosages (0, 0.2, 0.4 mmol/L) of PA, respectively, for 24 hours. And then, the 1 U/ml hCG was added into the petri dishes 4 hours early before the cell collection.

Furthermore, to assess the effect of CypD overexpression on the testosterone synthesis, cells were transfected with pLV‐PPIF (human CypD overexpression plasmid, Cyagen Biosciences Inc) using FuGENE^®^ HD Transfection Reagent as described by the manufacturer's instruction. The empty plasmids (pLV‐EGFP) were used as control. The media were changed after 8‐12 hours, and after 24‐hour culture, cells were collected for further analysis. Moreover, cells were treated with 0 and 0.4 mmol/L of PA for 24 hours, with or without a 1 hours pretreatment of cyclosporin A (CSA, an inhibitor of CypD, 1 μmol/L). Similarly, cells were collected for following analysis.

### 2.3 Detection of body and perineal fat distribution

To compare body fat distribution of whole body and perineum between the ND and HFD groups, X‐ray analysis was performed with Lunar Prodigy (GE Medical Systems, 8743, Madison, WI USA) according to the manufacturer's instruction. Mice were scanned under pentobarbital sodium anaesthesia. Scans were done through whole body, especially the perineum where the testes were located in.

### 2.4 Measurement of lipid profile and sex hormone

The mouse serum was separated after centrifugation. The concentration of lipid profiles containing triglyceride (TG), total cholesterol (TC), low‐density lipoprotein cholesterol (LDL‐C) and high‐density lipoprotein cholesterol (HDL‐C) was measured with biochemical analyser (Mindray Bio‐Medical Electronics Co., Ltd). Testosterone and luteinizing hormone (LH) levels were measured using enzyme‐linked immunosorbent assay (ELISA) kit according to the manufacturer's protocols.

Testicular tissues (20 mg) obtained from each mouse were homogenized by sonication in phosphate‐buffered solution (PBS, 200 μL) and then centrifuged at 10 000 *g* for 10 minutes. The supernatant was collected for intratesticular testosterone concentration assessment. For cells, at the end of the cell treatment, the culture media were concentrated by freeze dryers (Alpha 1‐2 LDplus, 17615, Germany) and then were melted with 50 μL of PBS per 60‐mm dish to assay the ability of testosterone synthesis and secretion of TM3 cells. The protein concentrations of tissues or cells were determined using BCA Protein Assay Kit (Shenneng Bocai). Testosterone concentrations were detected with ELISA kit for testosterone (Uscn Life Science & Technology Co., Ltd.) according to the kit manufacturer's protocol and then were normalized to protein concentrations.

### 2.5 Transmission electron microscope analysis

For transmission electron microscope analysis, fixed testicular sections were washed three times with phosphate buffer and were fixed in 1% osmium tetraoxide for 1 hour. The sections were dehydrated by a graded series of acetone and embedded in araldite. Ultrathin sections were cut with a diamond knife, stained with uranyl acetate and lead citrate, and viewed with an transmission electron microscope (JEM‐1200EX, JEOL Co., Japan).

### 2.6 Oil Red staining

To detect the lipid accumulation, the 5‐μm‐thickness frozen slices of testes and the slides of TM3 cells were stained with Oil Red and observed with the light microimaging system. Meanwhile, the area of Oil Red staining was quantified by ImageJ software.

### 2.7 RNA isolation and quantitative reverse transcription‐polymerase chain reaction (qRT‐PCR)

Total RNA of the testicular tissues or TM3 cells was extracted by using RNAiso Reagent, which subsequently was subjected to reverse transcription kit (PrimeScript^®^ RT Reagent Kit (Perfect Real Time), TaKaRa, Japan) according to the manufacturer's protocol and was transformed to cDNA with common PCR instrument (Eppendorf Scientific. Inc) at 37°C for 15 minutes. The cDNA was used as a template for qRT‐PCR conducted with the LightCycler480 (Roche). The cycling parameters were 95°C for 10 minutes, 40 cycles at 95°C for 15 seconds; 60°C for 60 seconds. The oligonucleotide primers used are shown in Table S1. Each specimen was analysed in triplicate. Transcript levels were normalized to those of β‐actin, and relative expression levels were calculated using the 2^–ΔΔCt^ method.

### 2.8 Immunoblotting analysis

Total protein was extracted from testicular tissues or Leydig cells with protein lysis buffer (RIPA: PMSF = 100:1), and concentration was subsequently determined. Protein sample (100 µg) was separated by 12% separating gel and electrophoretically transferred to polyvinylidene difluoride membranes (PVDF membrane, Millipore Corporate). The membranes were blocked with 5% skimmed milk in TBST buffer and then probed with primary antibodies: anti‐β‐actin (1:7500; 60008, Protein Tech.), anti‐StAR (1:400; Santa Cruz Biotechnology) and anti‐CypD (1:1000; ab155979, Abcam) overnight at 4°C. The membranes were subsequently incubated with secondary antibodies (1:5000; Sigma‐Aldrich). Finally, the bands were visualized and quantified by FluorChem Q (Protein Simple, USA). The relative expression levels of tested proteins were normalized to the corresponding β‐actin.

### 2.9 Immunofluorescence

For immunofluorescence, fixed tissues were heat treated in Tris‐EDTA buffer for antigen retrieval. After washed by PBS, sections were blocked with 5% goat serum albumin prior to the addition of the primary antibody: rabbit anti‐StAR (1:100), and then incubated overnight at 4°C. After that, paraffin sections were incubated with the FITC‐conjugated goat anti‐rabbit IgG antibody (1:50, Sigma‐Aldrich) for 1 hour at room temperature. The nuclei were stained with DAPI (Cat. C1006, Beyotime). After treatment at least five consecutive fields, each slice was observed with a fluorescence microscope (Axio Imager A2, Zeiss, Jena, Germany).

### 2.10 Cell Mito stress test

The mitochondria energy metabolism of TM3 cells was determined with Agilent Seahorse XF96 Extracellular Flux Analyzer (Seahorse Bioscience, Agilent Technologies). TM3 cells were seeded at a density of 6 × 10^3^ cells/well in XF96 Cell Culture Microplates. After 24 hours with PA (0, 0.2 and 0.4 mmol/L) treatment, cells were washed with XF base medium supplemented with 2.5 mmol/L glutamine, 0.05 mmol/L sodium pyruvate and 17.5 mmol/L glucose, in which pH was adjusted to 7.4 at 37°C, and then, cells were incubated in a CO_2_‐free incubator at 37°C for 1 hour. Seahorse Cell Mito Stress Test was performed to measure oxygen consumption rate (OCR) with a sequential addition of inhibitors of mitochondrial function: oligomycin (2 μmol/L), carbonyl cyanide‐p‐trifluoromethoxyphenylhydrazone (FCCP, 1 mmol/L) and a combination of rotenone and antimycin A (0.5 mmol/L). The mitochondrial OCR in basal respiration, ATP production, maximal and spare respiration was monitored with analyser. Results were normalized after adjustment for the corresponding total protein content per well.

### 2.11 Analysis of mitochondrial membrane potential (ΔΨm) with JC‐1

Changes of the mitochondrial membrane potential (ΔΨm) were detected by 5, 5′, 6, 6′‐tetrachloro‐1,1′,3,3′‐tetraethylbenzimidazole‐carbocyanide iodine (JC‐1) staining according to the manufacturer's direction of the mitochondrial membrane potential assay kit (Beyotime, China). When mitochondria are in a state of normal polarization, JC‐1 aggregates to emit red fluorescence. While when mitochondria are depolarized, the monomeric forms of JC‐1 produce green fluorescence. The switch from red to green fluorescence was regarded as a reliable indicator of a loss in ΔΨm. Leydig cells were cultured in six‐well plates and were treated with BSA (menstruum, 0 mmol/L) or PA (0.4 mmol/L). After treatment for 24 hours, Leydig cells were incubated with 1ml JC‐1 staining solution at 37°C for 20 minutes in the dark. After the cells were washed twice with freshly prepared JC‐1 staining buffer (1×), the fluorescence changes were detected with a fluorescence microscope (Axio Cam HRC, Zeiss, Germany). The wavelengths of excitation and emission were 490 and 530 nm for detection of monomeric forms of JC‐1, and 525 and 590 nm were used to detect aggregations of JC‐1.

### 2.12 Mitochondrial ATP assay

The ATP levels of Leydig cells were measured by using a firefly luciferase based on ATP assay kit (S0026, Beyotime) according to the manufacturer's instructions. The fluorescence luminance (RLU) was measured with the luminometer (Multimode Reader LB942 TriStar2, BERTHOLD, Germany). The unit of ATP was corrected to nmol/mg with concentration of mitochondrial protein.

### 2.13 Assay of reactive oxygen species generation in mitochondria

After treatment with PA in 96‐well plates, TM3 cells were washed with PBS three times. As per the manufacturer's direction, 100 μL MitoSOX working solution was added into each well and then the plate was placed in the incubator at 37°C away from light for 10 min. And then, mitotracker for the mitochondria detection presenting green fluorescence was added into the medium in proportion as 1:10 000. After 10‐minute incubation the same as the above, the working solution was removed and the plate was washed with PBS three times again. Next, every 100 μL of Hochest mixed solution with DMEM basic medium (1:10) for the cell nucleus detection revealed as blue fluorescence was added and the plate was placed in the incubator at 37°C away from light for 3 minutes. The fluorescence situation was observed with the fluorescence microscope.

### 2.14 Statistical analyses

Statistical analyses were conducted using GraphPad Prism (v.5, GraphPad Software Inc, CA). All samples were determined by an independent‐sample *t* test or one‐way analysis of variance (ANOVA) followed by the least significant difference (LSD) multiple comparison test. All data were expressed as mean ± SEM, and the difference was considered to be statistically significant if *P* < 0.05.

## 3 RESULTS

### 3.1 General conditions and lipid deposition in Leydig cells of mice with high‐fat intake

**Figure 1:**
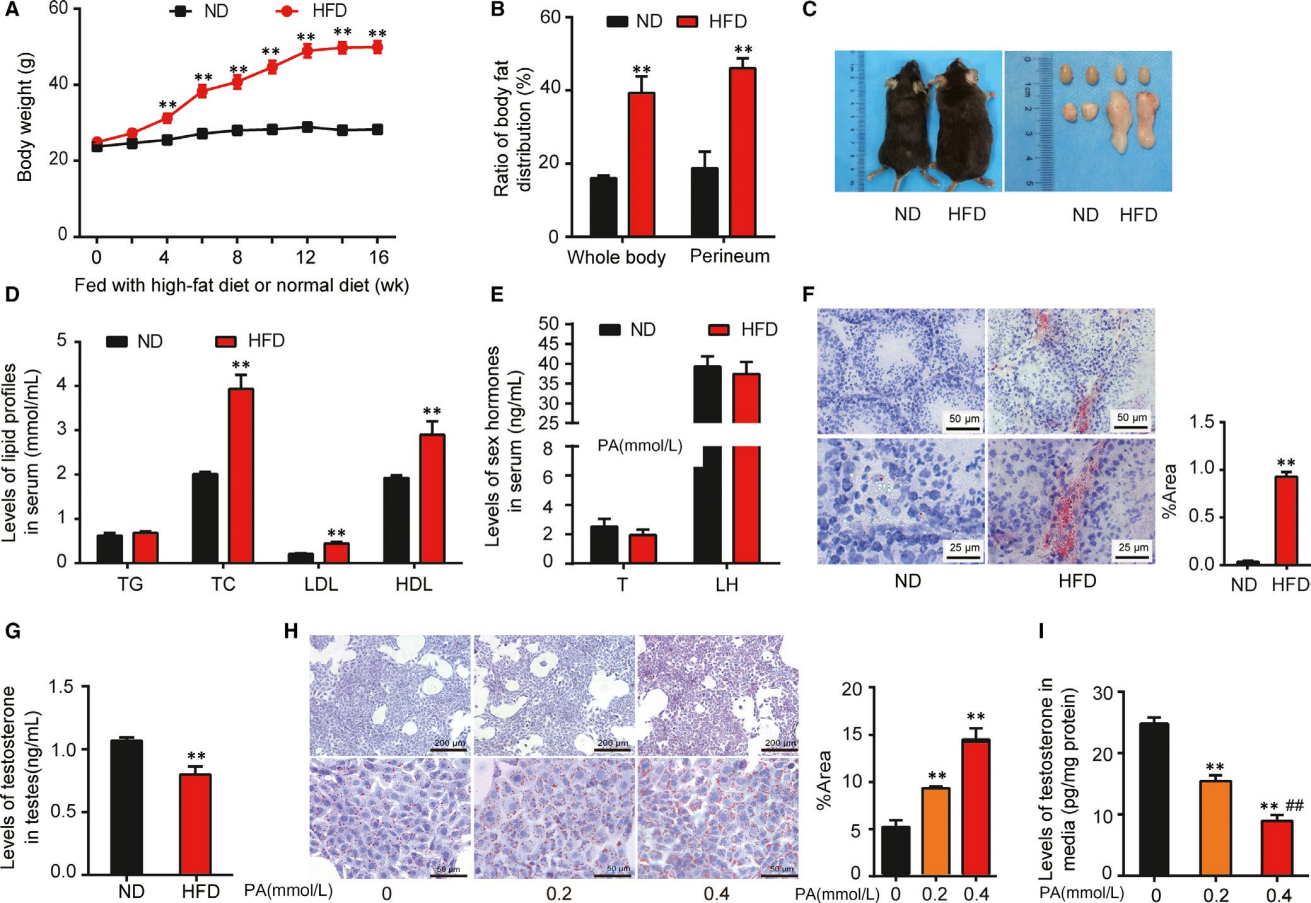
General conditions and lipid deposition in Leydig cells of mice with high‐fat intake. A, Comparison of bodyweight of mice fed with ND or HFD during 16 weeks. B, Ratio of fat distribution of whole body or perineum in the ND or HFD group at the 16th week of feeding. C, Representative photographs of somatotype, testes and epididymal fat of mice fed with ND or HFD at the end of the 16th week. D, Comparison of serum lipid levels between the ND and HFD groups. E, Comparison of serum sex hormone levels between the ND and HFD groups, including testosterone (T) and LH at the end of the 16th week. T: n = 10; LH: n = 5. F, Representative Oil Red staining sections and the area quantitative analysis showing lipid deposition of interstitial tissue of testes from mice in the ND or HFD group at the 16th week. G, Intratesticular testosterone levels of mice in the ND or HFD group. H, Representative Oil Red staining and the area quantitative analysis showing lipid accumulation in the Leydig cells with treatments of 0, 0.2 and 0.4 mmol/L of PA, respectively. I, Testosterone concentrations in media of TM3 cells exposed to 0, 0.2 and 0.4 mmol/L of PA for 24 hours. All data were represented as mean ± SEM, n = 4‐11 for each group. ^**^ *P* < 0.01 vs ND or 0 PA group, and ^##^ *P* < 0.01 vs 0.2 PA group were considered highly significant difference from the control

To analyse the effects of HFD on the testosterone levels, 8‐week‐old mice were given HFD or ND for 16 weeks. Compared with the ND group, the weight of mice in the HFD group increased significantly from the 4th week after being fed with high fat and continued to increase with the prolonged feeding time. By the 16th week, the average weight tended to be stable in the HFD group and it was 1.77 times of the ND group (*P* < 0.01; Figure 1A. The body fat distribution analysis with X‐ray further testified that the average percentage of body fat in perineum, around the testes, was 46.08% in the HFD group versus 18.70% in the ND group, showing the HFD group was 2.46 times of the ND group (*P* < 0.01, Figure 1B, consistent with the tendency of fat distribution of whole body as well as weight changes above. Correspondingly, representative photographs at the end of 16th week are shown in Figure 1C. The measurement of serum lipid profiles of mice displayed an obvious enhancement in the levels of TC (3.93 ± 0.10 vs 2.01 ± 0.01, *P* < 0.01), LDL‐C (0.44 ± 0.01 vs 0.21 ± 0.01, *P* < 0.01) and HDL‐C (2.90 ± 0.09 vs 1.93 ± 0.02, *P* < 0.01) in the HFD group in comparison with the ND group. The levels of TG in the HFD group were not statistically significant relative to the ND group Figure 1D. As shown in Figure 1E, serum sex hormone detection revealed no significant change in both testosterone and LH in the HFD group compared with the ND group.

In consideration of testicular Leydig cells as the main place to synthesize and secrete testosterone in male, the role of HFD on intratesticular testosterone levels was further assessed. Oil Red staining of the testes showed that the lipid accumulations were found mainly in the Leydig cells. Meanwhile, there was more visible lipid deposition in interstitial tissue of testes in the HFD group than that in the ND group and quantitative analysis supported the above conclusion (*P* < 0.01, Figure 1F. It was noticeable that there was an obvious decline in the intratesticular testosterone levels in the HFD group in contrast to the ND group (*P* < 0.01, Figure 1G).

A mass of PA, one of the main ingredients of high triglyceride diet, is responsible for lipotoxicity. In vitro, the TM3 Leydig cells were treated with different concentrations of PA (0, 0.2 and 0.4 mmol/L) and the effects on testosterone levels were analysed. The slides stained with Oil Red and quantitative analysis showed increased lipid accumulation in the TM3 cells exposed to PA in a dose‐dependent manner (*P* < 0.01, Figure 1H. With the increases in the PA concentration, there were obviously dose‐dependent decreases in the levels of testosterone secreted into the media by cells (*p*_0 vs. 0.2_ < 0.01, *P*_0.2 vs. 0.4_ < 0.01, Figure 1I).

### 3.2 High‐fat intake down‐regulates the expression of StAR in Leydig cells

**Figure 2:**
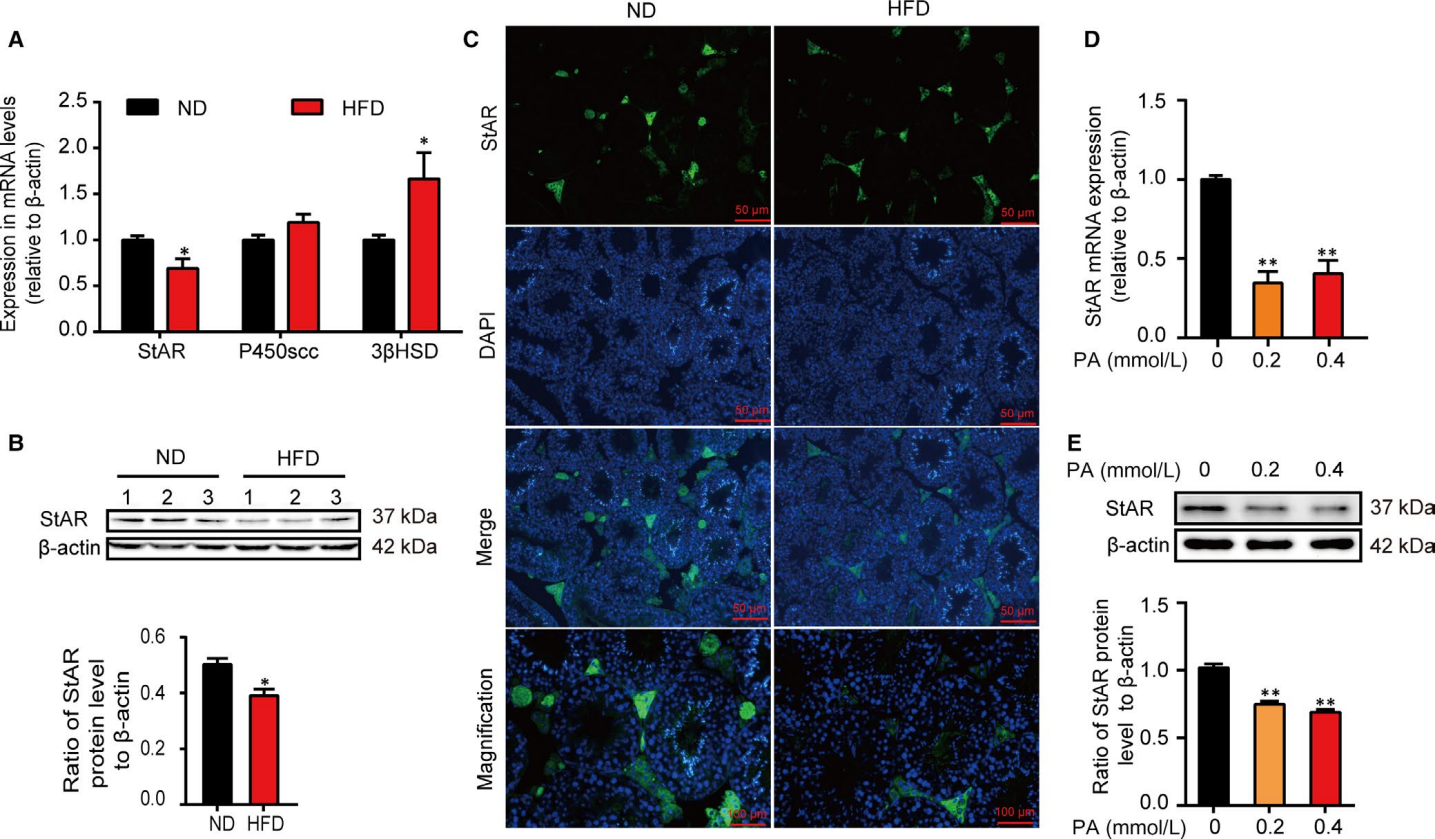
High‐fat intake down‐regulates the expression of StAR in Leydig cells. A, qRT‐PCR analysis of the expression of StAR, P450scc and 3β‐HSD mRNA in testes from mice in the ND or HFD group. B, Immunoblot and quantitative analysis of StAR protein in testes of mice in the ND or HFD group. C, Representative immunofluorescence assay showing the localization and quantitative analysis of StAR in testicular interstitial tissue from mice in the ND or HFD group. Green fluorescence represented StAR and blue fluorescence represented cell nucleus. D, qRT‐PCR analysis showing the relative expression of StAR mRNA and E, immunoblot and quantitative analysis of StAR protein abundance in TM3 cells treated with 0, 0.2 and 0.4 mmol/L of PA, respectively. Representative images for quantitative immunoblot are shown. ND, n = 3 and HFD, n = 3. Data were represented as mean ± SEM. ^*^ *P* < 0.05 and ^**^ *P* < 0.01 vs ND or 0 PA group were considered highly significant difference from the control

StAR assists the transportation of cholesterol from the OMM to the IMM, which is the first and rate‐limiting step in steroidogenesis process. And then, P450scc across the IMM catalyses cholesterol conversion to pregnenolone which next diffuses to the smooth endoplasmic reticulum where 3β‐HSD facilitates pregnenolone to form the more biologically active androstenedione, the immediate precursor of testosterone. All the above three proteins or enzymes play key roles during the steroidogenesis process. Herein, the effects of HFD on StAR, P450scc and 3β‐HSD were detected in mRNA levels firstly. Different from P450scc and 3β‐HSD, StAR mRNA relative expression was significantly declined in the HFD group in comparison with the ND group (*P* < 0.05; Figure 2A. StAR expression in the protein levels was assessed subsequently, which showed a statistically significant reduction in the HFD group than the ND group (*P* < 0.05, Figure 2B. Furthermore, immunofluorescence of testicular tissues indicated that, compared with the ND group, StAR in the Leydig cells decreased in the HFD group Figure 2C. Next, the influences of PA on StAR in Leydig cells were further analysed in vitro. After the treatment with 0, 0.2 and 0.4 mmol/L of PA, respectively, in the mRNA levels, there was significant decline in the 0.2 mmol/L or 0.4 mmol/L of the PA group relative to the 0 mmol/L of the PA group (*P* < 0.01, Figure 2D). Meanwhile, in the protein levels, in comparison with the control group, the expressions of StAR decreased significantly in the 0.2 mmol/L or 0.4 mmol/L group (*P* < 0.01, Figure 2E).

### 3.3 High‐fat intake has detrimental effects on the mitochondria in Leydig cells

**Figure 3:**
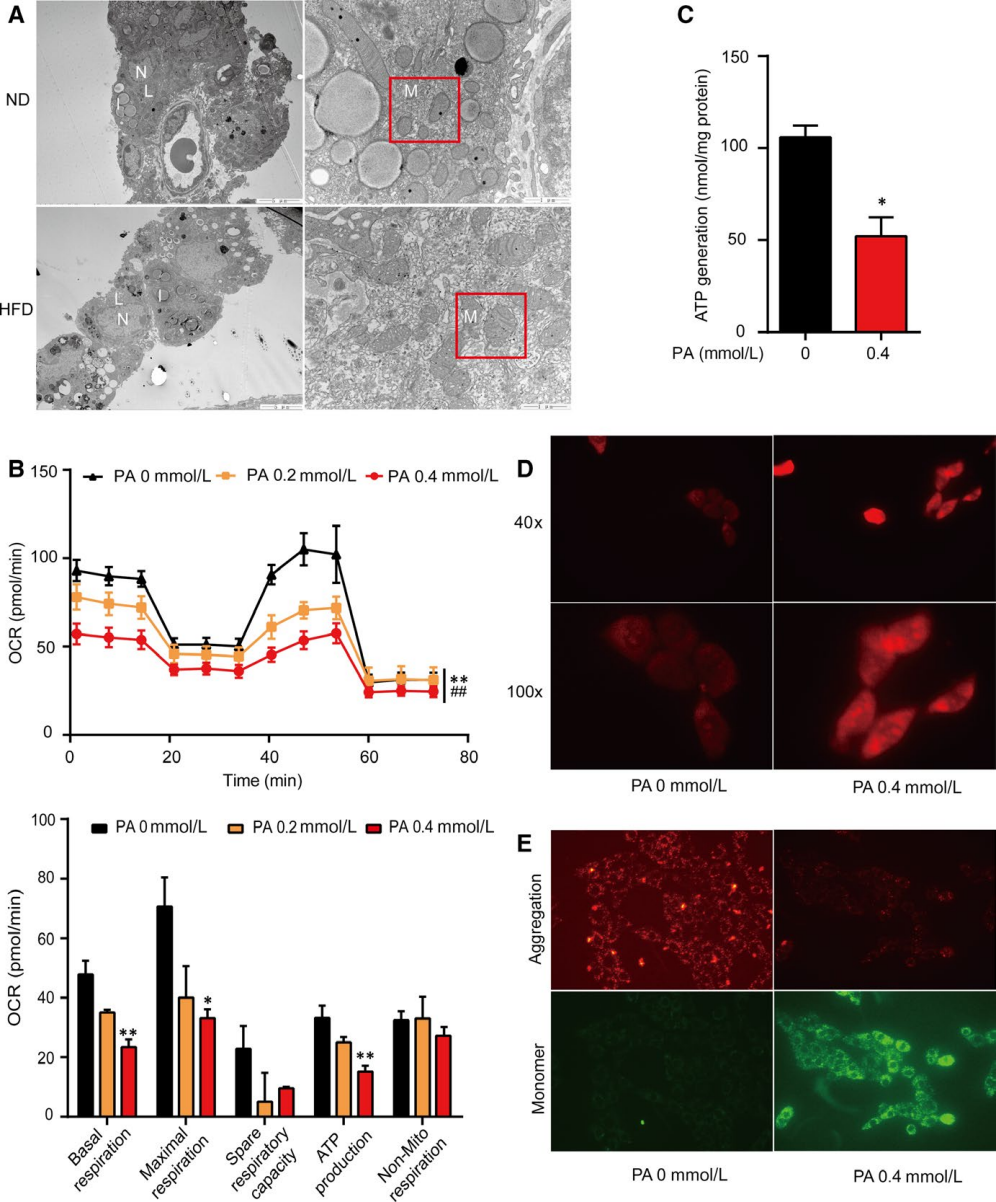
High‐fat intake has detrimental effects on the mitochondria in Leydig cells. A, Representative transmission electron microscopic images of Leydig cells of testes from ND and HFD mice showing Leydig cells (L), mitochondria (M), lipid droplets (l) with an high electron density and nucleus (N). B, Mitochondrial function detection via Seahorse XF Cell Mito Stress Test. C, ATP generation from mitochondria in 0 mmol/L or 0.4 mmol/L of the PA group. D, Measurement of ROS production by MitoSOX staining. Red fluorescence represented ROS. E, Determination of Δψm with the JC‐1 kit. Red fluorescence represented JC‐1 aggregations and green fluorescence represented JC‐1 monomers. Data were presented as mean ± SEM. ^*^ *P* < 0.05 and ^**^ *P* < 0.01 vs 0 PA group, ^##^ *P* < 0.01 vs 0.2 PA group were considered highly significant difference from the control

Mitochondria are the essential organelles of ATP synthesis as well as testosterone synthesis, which dysfunction would disturb StAR activity. We further examined the effects of HFD or PA on the mitochondrial function. First, representative electron micrographs of Leydig cells in testes showed that, compared with the ND group, there was an aberrant separation between the IMM and the OMM, and the intermembrane space was dilated mildly in some mitochondria in the HFD group Figure 3A. The FCCP Optimization with the XF Cell Mito Stress Test detecting mitochondrial energy metabolism showed that OCR in the PA group (0.2 or 0.4 mmol/L) declined in the dose‐dependent manner compared with the control group (0 mmol/L), suggesting that the mitochondrial function of the PA group was destroyed (*P* < 0.01, Figure 3B. Simultaneously, consistent with the detection of ATP in the Mito Stress Test, the ATP production measurement with kit displayed that ATP production of mitochondria in 0.4 mmol/L of the PA group was significantly lower than that in the control group (*P* < 0.05, Figure 3C. The results of MitoSOX staining showed that ROS production of the PA group was significantly more than that of the control group Figure 3D. It has been reported that StAR can play a synergistic effect on the cholesterol transport into the IMM only when the Δψ_m_ is normal. So, we detected whether PA affects the Δψ_m_ via JC‐1 assay. In comparison with the control group, the JC‐1 aggregations (red fluorescence) reduced while the JC‐1 monomers (green fluorescence) increased, indicating that the Δψ_m_ declined in the PA group Figure 3E.

### 3.4 High‐fat intake up‐regulates the expression of CypD in Leydig cells

**Figure 4:**
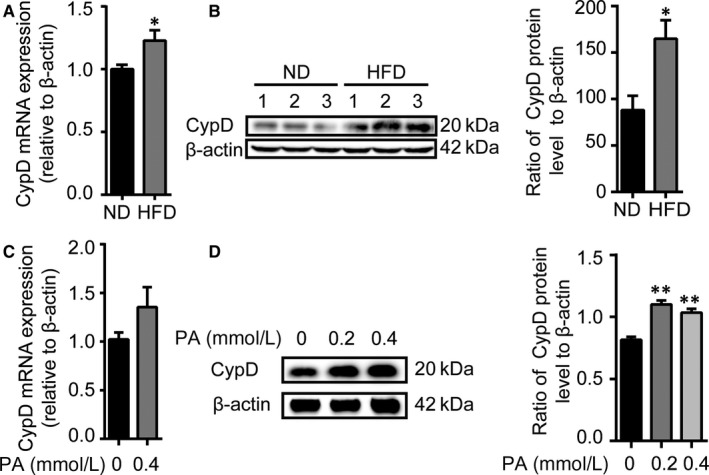
High‐fat intake up‐regulates the expression of CypD in Leydig cells. A, qRT‐PCR analysis of the relative expression of CypD mRNA and B, representative images for immunoblot and quantitative analysis of CypD protein abundance of testes of mice in the ND or HFD group. C, qRT‐PCR analysis showing the relative expression of CypD mRNA and D, representative images for immunoblot and quantitative analysis of CypD protein abundance in TM3 cells treated with 0, 0.2, 0.4 mmol/L of PA, respectively. Data were presented as mean ± SEM, n = 3 for each group. ^*^ *P* < 0.05 vs ND or 0 PA group was considered highly significant difference from the control

Under the condition of stress, the overly increase in CypD expression caused the exceedingly opening of mPTP followed with mitochondrial dysfunction, which had a detrimental effect on the StAR activity. In order to detect whether CypD is involved in the HFD‐mediated mitochondrial dysfunction, we further determined the expression of CypD of testes, respectively, in mRNA and protein levels. Compared with the ND group, CypD expression in mRNA levels increased with significant statistical difference (*P* < 0.05; Figure 4A). Similarly, in protein levels, CypD expression in the HFD group was up‐regulated and there was a statistical difference in the ratio of CypD protein level to β‐actin compared with the ND group (*P* < 0.05, Figure 4B). Although in vitro the increase in CypD mRNA expression of TM3 cells was not statistically significant (*P* > 0.05, Figure 4C), the protein expression of CypD in the PA‐treated group (0.2 or 0.4 mmol/L) was enhanced in contrast to the control group (*P* < 0.01, Figure 4D).

### 3.5 CypD overexpression down‐regulates expression of StAR

**Figure 5:**
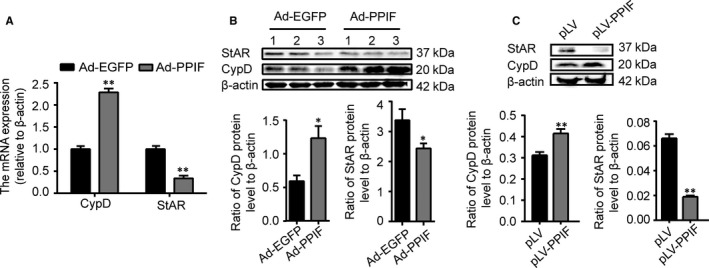
CypD overexpression down‐regulates expression of StAR. A, qRT‐PCR analysis of the relative expression of CypD and StAR mRNA and B, representative images for immunoblot and quantitative analysis of CypD and StAR protein abundance of testes of mice in the Ad‐EGFP or Ad‐PPIF group. C, Representative images for immunoblot and quantitative analysis of CypD and StAR protein abundance of TM3 cells in the pLV and pLV‐PPIF group. The ratio of CypD and StAR protein level to β‐actin was calculated. Data were presented as mean ± SEM, n = 3 for each group. ^*^ *P* < 0.05 and ^**^ *P* < 0.01 vs Ad‐EGFP group were considered highly significant difference from the control

To further testify the role of CypD in regulating StAR expression, StAR expression was analysed after overexpression of CypD in vivo and in vitro. The CypD overexpression mouse model was made with Ad‐PPIF by caudal vein injecting. As shown in Figure 5A, with increasing expression of CypD mRNA in testes, StAR mRNA levels decreased (*P* < 0.01). The levels of protein expressions were consistent with its mRNA levels (*P* < 0.05, Figure 5B). Then, the use of pLV plasmid containing CypD cDNA (*Ppif*) in vitro definitely caused the up‐regulation of CypD in protein levels along with the down‐regulation of StAR expression in contrast to the control group (*P* < 0.01, Figure 5C. All the above results indicated that CypD overexpression was negatively correlated with StAR expression at both mRNA and protein levels.

### 3.6 Inhibition of CypD improves the inhibiting role of lipotoxicity on the StAR

**Figure 6:**
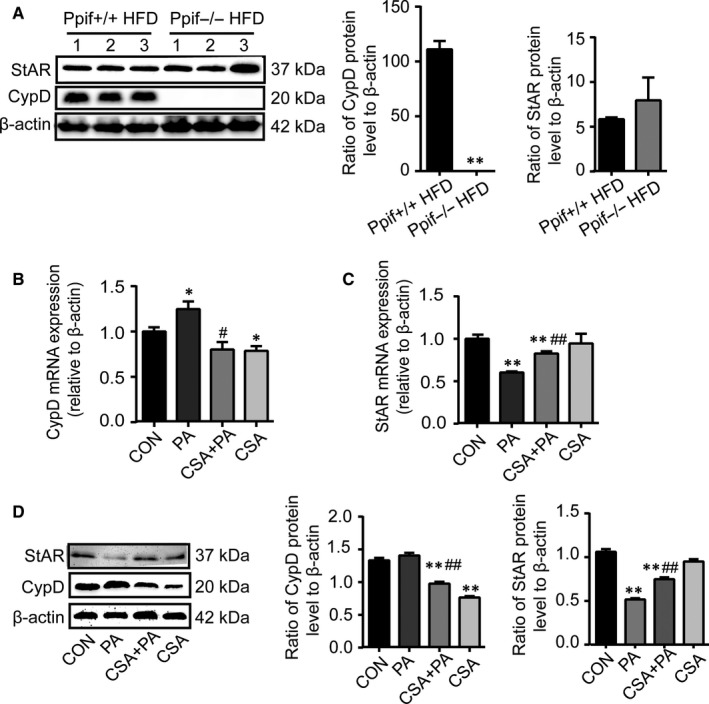
Inhibition of CypD improves the inhibiting role of lipotoxicity on the StAR. A, Representative images for immunoblot and quantitative analysis of CypD and StAR protein abundance of testes of *Ppif* ^+/+^ or *Ppif* ^−/^ *^−^* mice with HFD (n = 3). B and C, qRT‐PCR analysis of the relative expression of CypD and StAR mRNA of TM3 cells exposed to PA with or without the CSA pretreatment. D, Representative images for immunoblot and densitometry analysis of CypD and StAR protein of TM3 cells exposed to PA with or without the CSA pretreatment. Data were represented as mean ± SEM. ^*^ *P* < 0.05 and ^**^ *P* < 0.01 vs ppif^+/+^ HFD or control group, ^#^ *P* < 0.05 and ^##^ *P* < 0.01 vs PA group were considered highly significant difference from the control

To clarify whether HFD‐induced CypD overexpression is a key point in the process of down‐regulation StAR, the CypD complete knockout (*Ppif* ^−/−^ *)* mouse model was applied in this study. In Figure 6A, the immunoblot revealed that, in comparison with the *Ppif^+/+^* HFD group, the CypD in the *Ppif* ^−/−^ HFD group was certainly inhibited totally while the StAR in protein level has no significant difference, suggesting that HFD could not down‐regulate StAR expression in the context of CypD deficiency.

The above assumption was further examined in vitro. It has been reported that cyclosporin A (CSA) was one of the nonspecific inhibitors on the mitochondrial oxidation and disturbed the opening of the mPTP. It was used to demonstrate whether CSA could alleviate PA‐induced StAR down‐regulation by inhibiting the CypD expression in Leydig cells. As shown in Figure 6B and 6D, the mRNA and protein expressions of CypD in CSA pretreatment group were less than those in the control group, indicating that CSA inhibited CypD expression, while both mRNA and protein levels of StAR did not show differences between the control group and pretreatment group Figure 6C and 6D, suggesting that CSA alone has no influence on StAR. Interestingly, in the CSA + PA group in which TM3 cells were pretreated with CSA and subsequently exposed to PA, the protein level of CypD was less than that in the control group and was similar to that in the CSA group, showing that PA could not elevate again CypD expression inhibited by CSA in the protein level. In the same time, although both mRNA and protein levels of StAR in the CSA + PA group were still lower than the control group, its expression levels increased with statistical difference in comparison with the PA group Figure 6C and 6. These results indicated that blocking of CypD could ameliorate the PA‐induced down‐regulation of StAR expression and demonstrated that CypD played an indispensable role in this process.

## 4 DISCUSSION

Clinical studies suggested a decreased testosterone level in serum in reproductive‐age men with diet‐induced obesity.^31^, ^32^ However, the underlying mechanisms are still not obvious. The Leydig cells, which are located between the seminiferous tubules of the testis, are responsible for the synthesis and secretion of testosterone. Our previous studies have reported that dietary factors such as high‐cholesterol diets could inhibit Leydig cell steroidogenesis.^33^ In this study, we demonstrated that the high‐fat diet could induce lipid accumulation in testicular Leydig cells. Subsequently, over‐activated CypD induced mitochondrial dysfunction, accompanied by a decrease in StAR expression, which is the rate‐limiting step of the testosterone biosynthesis, resulting in reduced testosterone production. Importantly, blocking CypD overactivation with CSA or knocking out CypD can alleviate the abnormal expression of StAR in Leydig cell induced by high‐fat diet.

To investigate the effects of lipid overload on testicular Leydig cells, the HFD mouse model was used to show that the weight of mice in the HFD group increased obviously, accompanied by dyslipidaemia and increased adipose tissue. In this model, diet‐induced dyslipidaemia, characterized by rising in TC, LDL‐C and HDL‐C, was in line with the study of Fan Yong et al.^9^ Even in these animal experiments, there was no significant difference in TG levels, and these above results still demonstrated the success of the construction of HFD mouse model simulating a similar human condition. Some studies describing the hormonal abnormalities in the hypothalamic‐pituitary‐gonadal (HPG) axis in obese men indicated that obese men had normal LH levels with decreased total testosterone levels.^34^, ^35^, ^36^, ^37^ Giagulli et al 38 further demonstrated that moderately obese men (BMI 30‐35 kg/m^2^) had normal LH pulsatility, while morbidly obese men (BMI > 40 kg/m^2^) had decreased LH levels and LH amplitudes. In our study, there were no significant differences of the serum testosterone and LH levels between groups. One of the reasons might be that HFD mouse model has yet not attained the morbid obesity extent. Under physiological conditions, the levels of intratesticular testosterone (ITT) in male were some 40‐ to 100‐fold higher than those in the circulation.^39^, ^40^, ^41^ So, the concentrations of ITT were measured subsequently. The results suggested an evident decline of ITT in the HFD group relative to the ND group. Meanwhile, the Oil Red staining of testicular tissues demonstrated fat accumulation in the interstitial tissue of testes. The detections of corresponding index of TM3 cells with PA treatment showed consistent conclusions with the animal experiments. These results intuitively indicated that high‐fat intake reduced ITT levels, accompanied by lipid accumulation in testicular Leydig cells.

During the process of testosterone biosynthesis in Leydig cells, the extra‐mitochondrial 37‐kDa StAR precursor facilitates the transfer of cholesterol into the mitochondrial matrix,^42^ known as the initiating and rate‐limiting step in steroidogenic process. Meanwhile, the 37 kDa precursor also enters the mitochondrial matrix together and is further transformed to a 30 kDa form. The rate‐limiting enzyme StAR which is vital in the process is located on the OMM. In our study, quantitative RT‐PCR analyses showed an apparent descent in the mRNA levels of StAR in the HFD group compared with the ND group, illustrating long‐time HFD could reduce the testosterone levels through suppression on StAR transcription. Subsequently, the quantization and localization of StAR protein were, respectively, monitored by immunoblot and immunofluorescent staining and the results further testified the inhibiting effect of long‐time HFD on StAR expression in Leydig cells. Simultaneously, in vitro TM3 Leydig cell exposure to different concentrations of PA showed down‐regulation in both StAR mRNA and protein levels in a dose‐dependent manner, consistent with the results in vivo.

Mitochondrial homeostasis is essential to maintain the expression and activity of StAR. Leydig cells are particularly sensitive to ROS produced by oxidative stress.^43^, ^44^ Previous researches indicated that the dissipation of mitochondrial membrane potential (∆Ψm) could cause StAR degradation. The decline in mitochondrial ATP levels reduces StAR translation. Moreover, ATP is important for StAR phosphorylation which is the way to enhance its activity and stability.^45^, ^46^ Therefore, we further detected whether long‐time HFD damaged mitochondrial functions. The representative transmission electron microscope (TEM) images of testes displayed a slight separation between IMM and OMM in the HFD group relative to the ND group, suggesting that long‐time HFD could cause the abnormal mitochondrial membrane structure or even broken. The reason may be that the increased permeability of IMM allowed some small molecules to permeate into the intermembrane space mildly. A series of experiments of TM3 cells with PA treatment were performed to further testify influences of PA on the mitochondrial function. The results of FCCP Optimization with the XF Cell Mito Stress Test showed dose‐dependent declines in oxygen consumption rate, maximal respiration, spare respiratory capacity and ATP production, suggesting that the mitochondrial function was disrupted in a dose‐dependent manner with PA treatment. Subsequently, Mito SOX Red staining was given to measure the concentrations of ROS in mitochondria, JC‐I was used to detect ΔΨm and mitochondria were isolated from TM3 cells to evaluate the level of ATP. TM3 cells showed consistent results with Mito Stress Test. In brief, these results both in vivo and in *vitro* demonstrated that prolonged HFD or PA led to mitochondrial dysfunction including reduced mitochondrial ATP production, increased ROS and dissipating ∆Ψm, which could down‐regulate the expression of StAR.

It was reported that prolonged HFD could impair the structure and function of mitochondria via up‐regulating the expression of CypD which was an essential regulator of mPTP.^30^ The mPTP is central to mitochondrial function. Persistent opening of the mPTP could lead to the dissipation of ∆Ψm, inactivation of ATPase, inhibition of the respiratory chain, rupture of OMM, and even apoptosis or cell death. Researches in CypD‐deficient and overexpression mice have shown that CypD plays a crucial role in the process of permeability transition (PT). In this study, the overexpression of CypD in both mRNA and protein levels of testes in mice from the HFD group was detected compared with the ND group and the results were consistent with the observations in TM3 cells treated with PA, indicating that long‐time high‐fat intake could elevate the CypD expressions in both mRNA and protein levels in a dose‐dependent manner.

Moreover, the hypothesis that long‐lasting HFD inhibits the StAR expression through CypD overexpression‐dependent mitochondrial dysfunction remains to be determined. Towards this end, we established two kinds of mouse models with either overexpressing CypD or deficient in CypD. The CypD overexpression mice were established by injecting Ad‐PPIF and Ad‐EGFP, respectively, via the caudal vein for 3 weeks. Both immunoblot and qRT‐PCR manifested that compared with mice with Ad‐EGFP, the CypD expression of mice with Ad‐PPIF increased, verifying the success of establishment of CypD overexpression mice, whereas the StAR expression of mice with Ad‐PPIF deceased, verifying the insights that the overexpression of CypD down‐regulated the StAR mRNA and protein expression levels. The results of relative detections in TM3 cells transfected with plasmid containing CypD were similar to those in vivo, supporting consistent insights into the inhibiting influences of overexpression of CypD on the StAR. The *Ppif* ^+/+^ mice and *Ppif^−^* ^/^ *^−^* mice were bred by sexual hybridization of *Ppif* ^±^ male and female mice. *Ppif^−^* ^/^ *^−^* mice were born at the expected Mendelian frequency, developed normally, and did not have any detectable phenotype and viability anomalies.^25^, ^26^ We observed that there was apparent increase of StAR expression levels in *Ppif^−^* ^/^ *^−^* mice in the HFD group compared with those in *Ppif* ^+/+^ mice in the HFD group, suggesting that the CypD through deletion could help StAR to resist to long‐standing HFD down‐regulated effects. Simultaneously, the pretreatment with CSA before PA treatment was performed to TM3 cells and the results indicated that the inhibition of CypD via CSA could alleviate PA noxious implication on the StAR expression. We therefore identified a possible mechanism that ablation of CypD attenuated the HFD‐induced StAR down‐regulation. However, the concrete mechanism through which CypD overexpression inhibits StAR expression is unclear and needs further investigation.

In conclusion, the present study demonstrated that CypD was involved in inhibition of StAR expression and activity by HFD. However, there were still some deficiencies in this study. In the present study, the CypD overexpression model was performed by tail vein injection of Ad‐PPIF, and the knockout model was global gene knockout, in which we could not rule out the influences of holistic factors. It has been reported that the roles of CypD in metabolic homeostasis were tissue‐specific.^21^ In the future, the Leydig cell‐specific deletion of CypD models will be used. Moreover, how CypD regulates the mPTP switch in Leydig cells still needs to be further explored. CypD plays important roles in tissue ischaemia‐reperfusion injury, apoptosis and metabolic homeostasis in some cells; therefore, the studies of roles of CypD in testosterone synthesis as well as in metabolic homeostasis in Leydig cells could provide therapeutic target for HFD‐induced testosterone deficiency.

## CONFLICT OF INTEREST

There are no conflicts of interest to declare.

## AUTHOR CONTRIBUTIONS

Y. C. and GQ designed the experiments. SX, LD and ZY performed experiments, testing and data analyses. YC and WX provided technical expertise. YC and SX wrote the manuscript. SX and WX organized the mice. X.X measured levels of testosterone and LH. SM and YJ administrated mice fed with HFD or ND. LD and ZM provided cell treatment skills. All authors edited the manuscript and provided comments.

## Data Availability

The data used to support the findings of this study are available from the corresponding author upon request.
