# Supplementary material for: Cyclophilin D participates in the inhibitory effect of high‐fat diet on the expression of steroidogenic acute regulatory protein
Source: J Cell Mol Med. 2019 Aug 1;23(10):6859–71. doi: 10.1111/jcmm.14569 (PMC6787510; doi:10.1111/jcmm.14569)
Supplement: Supplementary file 1 [file JCMM-23-6859-s001.docx]

**Supplementary Table 1** Murine primer sequences for qRT-PCR.

| **Gene** | **NM** |  | **Forward Primer** | **Reverse Primer** |
| --- | --- | --- | --- | --- |
| StAR | NC_000074.6 |  | ATGTTCCTCGCTACGTTCAAG | CCCAGTGCTCTCCAGTTGAG |
| CypD | NC_000080.6 |  | TCACCCAGGTTCTCCAAA | AGAAACGGGATCTGAGGC |
| P450scc | NC_000075.6 |  | AGGTCCTTCAATGAGATCCCTT | TCCCTGTAAATGGGGCCATAC |
| 3β-HSD | NC_000069.6 |  | AGCTCTGGACAAAGTATTCCGA | GCCTCCAATAGGTTCTGGGT |
| β-actin | NC_000071.6 |  | GGCTGTATTCCCCTCCATCG | CCAGTTGGTAACAATGCCATGT |
